# Are there Reliable Qualitative Individual Differences in Cognition? Probably Not

**DOI:** 10.5334/joc.174

**Published:** 2021-08-27

**Authors:** Claudia C. von Bastian

**Affiliations:** 1 Department of Psychology, University of Sheffield, Cathedral Court, 1 Vicar Lane, Sheffield S1 1HD, UK

**Keywords:** Individual Differences, Attentional Control, Psychometrics

## Abstract

Rouder and Haaf (2021, this issue) propose a focus shift toward qualitative individual differences in cognition and present a research toolkit for doing so. In this invited commentary, I will argue that the observation of qualitative individual differences may be no more than an indicator that more theoretical and empirical work needs to be done to identify the mechanisms and abilities underlying these individual differences in directionality. I will then move on to discuss how the toolkit can be used though to investigate one of the currently most central current topics in cognitive differential psychology, that is, the question over the existence of true quantitative individual differences in attentional control experimental effects. I conclude that, while highly valuable, no toolkit can save us from facing the challenging theoretical and conceptual questions in this area.

In their article, Rouder and Haaf (2021) propose distinguishing between qualitative and quantitative individual differences in cognition, and they introduce a user-friendly toolkit for evaluating empirical evidence accordingly. Qualitative individual differences occur if people differ in the direction of an experimental effect. As an example, Rouder and Haaf discuss that although most people show a rightward bias in an orientation discrimination task, some people show a leftward bias. Quantitative individual differences occur if all people show an experimental effect in the same direction. For example, they empirically demonstrated that everybody shows a Stroop effect, that is, reacts more quickly and correctly to trials that are congruent (i.e., ink color and label match) than to trials that are incongruent (i.e., ink color and label do not match). A third possibility is that individual differences in an experimental effect are negligible, that is, everybody will show the same effect to the same extent.

## Do Irreducible Qualitative Individual Differences Exist?

In their title, Rouder and Haaf (2021) pose the question of whether there are reliable qualitative individual differences in cognition. This question resonates well with some of the key questions in cognitive psychology that evolve around the directionality of individual differences. For example, does being bilingual yield cognitive advantages, disadvantages, or null effects? Does acquiring strategies that enhance performance in one task benefit, harm, or not affect performance at all in other tasks with different task structures and stimulus materials? Arguably, however, the implied directionality of these questions mainly exists in an experimental psychology framework in which groups or conditions are contrasted. From a differential psychology perspective, this question of directionality will then become a question of quantitative individual differences by shifting the focus toward investigating possible mediators and moderators of the experimental effect. For example, when observing qualitative individual differences in the effects of bilingualism in a particular effect, the natural next step in research would be to identify which quantitative individual differences – processing mechanisms and/or abilities – underlie these individual differences in directionality of the effect. Indeed, Rouder and Haaf suggest that the “ability to reduce qualitative individual differences to quantitative ones represents knowledge that informs theory, and doing so should be a general goal of psychological sciences” (p. 4), and that the quest to explain qualitative individual differences by “quantitative individual differences in rich processing mechanism” is “perhaps most exciting” (p. 12). The cross-race bias cited by Rouder and Haaf is an excellent example for reducible qualitative individual differences; I would suspect an explanation on a similar level can also account for the orientation effect presented as an example for true qualitative individual differences in cognition. In other words, observing qualitative individual differences may simply suggest that more theoretical and empirical work needs to be done in order to understand an experimental effect. Therefore, in this sense I agree with Rouder and Haaf in that what they dub the “arbitrary behavioral diversity hypothesis” is not an endpoint, but rather presents a starting point for further inquiry.

## Do Experimental Effects Without Individual Differences Exist?

A second question that can be addressed with Rouder and Haaf’s (2021) proposed toolkit is whether there are any true quantitative individual differences in a given experimental effect. For example, after having established that everybody shows a positive Stroop effect, do people differ in the extent of this effect, or does individual variation in this effect represent no more than noise? Indeed, a currently heatedly debated question centers around the existence of psychometrically sound individual differences in attentional control (see von Bastian et al., 2020). Attentional control, also referred to as executive functions or cognitive control, has been conceptualized as the ability to regulate information processing during goal-related behavior (e.g., Miyake et al., 2000). Individual differences in attentional control are thought to emerge in experimental effects when contrasting conditions with high attentional control demands (e.g., incongruent trials in the Stroop task, or switch trials in a mental set shifting task) with conditions with low attentional control demands (here, congruent trials in the Stroop task and repetition trials in a shifting task). However, weak correlations between such measures of attentional control (e.g., Paap & Greenberg, 2013; Rey-Mermet, Gade, & Oberauer, 2018; von Bastian et al., 2016) and low reliabilities of some of these tasks (e.g., Hedge, Powell, & Sumner, 2018; Paap & Sawi, 2016) have led researchers to call into question the current conceptualization and measurement of attentional control (e.g., Draheim, Tsukahara, Martin, Mashburn, & Engle, 2021, Rey-Mermet, Gade, Souza, von Bastian, & Oberauer, 2019, Rouder & Haaf, 2019).

Rouder and Haaf (2019) suggested that trial-to-trial noise obscures true individual differences in the typically rather small experimental effects in attentional control tasks and, thereby, may explain the low reliabilities and weak correlations. In their toolkit, Rouder and Haaf provide a simple function to account for trial noise, which is a highly welcome and useful tool that will make hierarchical modeling to account for trial noise accessible for researchers in this area. Now, the question is: can accounting for trial noise resolve the debate over the existence of attentional control as a sound psychometric construct? It is certainly helpful for demonstrating the existence of reliable individual variation in attentional control effects such as in the Stroop data from von Bastian et al. (2016) and Rey-Mermet et al. (2018). Still, the between-task correlations of the now more reliable measures remained low (Rouder, Kumar, & Haaf, 2019). Moreover, even if we were to observe higher correlations between these measures, the next important question would be that of measurement purity: Do the observed, now dis-attenuated correlations truly reflect quantitative individual differences in attentional control, or are they conflated with individual differences in other abilities? In the case of the assessment of attentional control, one such potential confound is general processing speed. This may become clearer with the following simple example: A generally slower person may complete congruent trials on average in 1000 ms, and incongruent trials in 1500 ms. The Stroop effect for this person can now be computed as the absolute difference between the two conditions, 1500–1000 = 500 ms, or as the proportional difference, (1500–1000)/1000 = 0.5. A generally faster person may complete congruent trials in just 500 ms, and incongruent trials in 750 ms. This would yield a much smaller absolute Stroop effect of only half the size – 250 ms – but an identical proportional effect of 0.5. Correlations between absolute differences will include individual differences in processing speed, which are accounted for when using proportional differences. Therefore, in addition to trial noise, it is critical to account for these differences in general processing speed to get to the bottom of the psychometrics of attentional control. In our own data, we found that these proportional experimental effects do correlate well for some facets of attentional control like mental set shifting (von Bastian & Druey, 2017) but not for inhibitory control tasks (e.g., De Simoni & von Bastian 2018; Guye & von Bastian, 2017). Accounting for general processing speed when assessing attentional control is central to the conceptualization of the construct. Specifically, where do we draw the line between processing speed and attentional control (see also Jewsbury et al., 2016)? Do we consider these to be two qualitatively different abilities, or are we happy with blurring the lines and considering them to be just two facets of the same underlying ability?

Taken together, trial noise alone fails to explain the inability to establish a psychometrically sound attentional control ability, and the question of how to derive measures for investigating individual differences in attentional control eventually becomes a theoretical-conceptual question more so than an analytical one. No toolkit can save us from addressing these, perhaps sometimes rather inconvenient, theoretical questions, but it can certainly help us in testing theoretical predictions of competing models of human cognition.
